# Automatic Optical Inspection System for Wire Color Sequence Detection

**DOI:** 10.3390/s22155885

**Published:** 2022-08-06

**Authors:** Tsung-Han Tsai, Chun-Sheng Cheng

**Affiliations:** Department of Electrical Engineering, National Central University, Taoyuan 32001, Taiwan

**Keywords:** automated optical inspection, color recognition, computer vision, surveillance system

## Abstract

Given the huge demand for wire in today’s society, the quality of the wire is especially required. To control the quality of the produced wire, the industry has a great desire for automated optical inspection technology. This technology is a high-speed and highly accurate optical image inspection system that uses mechanical sensing equipment to replace the human eye as the inspection method and simulates manual operation by means of a robotic arm. In this paper, a high-performance algorithm for the automated optical inspection of wire color sequence is proposed. This paper focuses on the design of a high-speed wire color sequence detection that can automatically adapt to different kinds of wires and recognition situations, such as a single wire with only one color, and one or two wires covered with aluminum foil. To be further able to successfully inspect even if the wire is short in the screen and the two wires are close to each other, we calculate the horizontal gradient of the wires by edge detection and morphological calculation and identify the types and color sequences of the wires in the screen by a series of discriminative mechanisms. Experimental results show that this method can achieve good accuracy while maintaining a good computation speed.

## 1. Introduction

Compared to high-tech industries with much professional equipment, traditional manufacturing factories rely on a large amount of manual labor, which is scattered throughout the production line to maintain the requirements of mass production. In production lines, the inspection stage is a relatively non-technical but unavoidable part of the process. In the current method, the operator usually observes the condition of the finished product through optical instruments or directly through the eyes. However, the human body accumulates fatigue due to long hours of work or various reasons that lead to a decrease in concentration, so relying on the human eye alone to judge is not rigorous enough.

For this reason, many manufacturers have partnered with technology companies to develop and produce a variety of automated equipment to replace labor-intensive manual operations, among which Automated Optical Inspection (AOI) is a high-speed, high-accuracy optical image inspection system. In factories equipped with vision sensor equipment, AOI is used to detect, judge, and filter out products with defects, and is widely used in various automated production as an improvement to the traditional human inspection using optical instruments. Especially at this time when most of the factories have moved into Industry 4.0, AOI is an indispensable part of it. A machine with AOI can operate automatically to save manpower on some decisions. The benefits of AOI are reliability and consistency. It shows great ability to integrate with the industrial Internet of Things (IoT) applications. With this system integration on AOI and IoT, many techniques are investigated to support this trend and finally to apply in products. However, different products have different inspection methods on AOI, so it is necessary to use the proper techniques to design the dedicated product.

**Figure 1:**
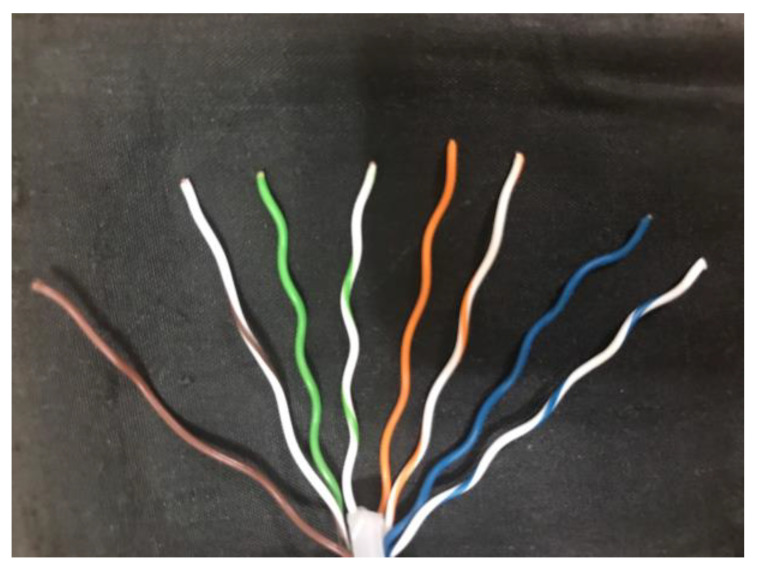
Different types of wire in RJ45 Ethernet connector.

In today’s society, there is a huge request for wire, such as cell phones, computers, and other consumer electronic products. While manufacturing in large quantities, the quality control of wire is very important. To ensure the quality of the finished product, many manufacturers require their employees to check the condition of the wire between processes, such as whether the wire has defects or is correctly aligned. Wiring is one of the more important steps in the manufacture of wire. At this stage, the wire is placed in the slot of the jig in the given color sequence. However, different types of wire have different color compositions. For example, Figure 1 shows the front end of RJ45 Ethernet connector. Since the next stage requires the placement of the arranged wires in a special mold for fixing, this step is irreversible. Once the formed product has any defects or wrong color arrangement, the whole product should be eliminated.

To check whether the color sequence of the wire is correct, the traditional method is to manually use a high-resolution digital microscope to zoom in directly, so self-inspection through the human eye alone still results in the waste of many wires. Among the existing methods, deep learning-based methods [1,2,3] are more mainstream to solve such automated optical inspection problems. This method trains the designed neural network model by giving a large amount of data, and the parameters are continuously updated to achieve higher accuracy. However, the large datasets required by deep learning-based methods are not easily available, and the complexity of training increases the cost of hardware and inspection time. For automatic optical inspection, real-time is extremely important. If the developed inspection system is slower than the original manual process, it can seriously slow down the production line of the factory. Compared to training neural network models, traditional feature extraction methods [4,5,6] have relatively low hardware requirements and fast computational speed but are less robust to the environment.

Since most wire factories place wires according to a uniform standard to identify the color sequence of the wires, the environmental impact is not significant. For the above reason, a system for detecting the color sequence of wires in a factory does not necessarily require a large neural network. In this paper, a system for identifying the color sequence of wires based on the traditional feature extraction method is proposed. Since the object to be detected is a wire in a clean background, edge detection is used to obtain edge characteristics. In addition, labels and specially designed methods are used to eliminate skin reflections and wrinkles that often occur when capturing wire. Finally, by using the designed identification method, the common types of wires are identified and then the color sequence from left to right is recognized. To match the requirements of most manufacturers, the proposed approach has the following contributions:

1. Identifying three common types of wire with specially designed color identification methods.
2. Using feature acquisition method to improve overall accuracy and maintain real-time processing.
3. Using a specially designed noise reduction method to eliminate light reflections and surface folds that are more difficult to remove.

## 2. Related Works

As human resources gradually failed to satisfy the quality requirements of companies, automatic optical inspection technology to replace human eyes and hands came into being. The core principle of automatic optical inspection technology depends on the use of optical instruments as inspection tools, and the difficulty is how to process the high-value optical images through the designed algorithms. In addition, there is a corresponding solution for different detection objects. In the following, existing technologies related to automatic optical inspection are discussed first, and the main architectures are divided into methods using convolutional neural networks (CNNs) and methods based on traditional feature extraction. After that, some common types of wire used today are explained.

### 2.1. Methods of Convolutional Neural Networks

Deep learning-based methods are relatively new and accurate techniques, among which transfer learning [7,8,9] has better adaptability to automatic optical techniques. For common machine learning techniques, the learning system can only process samples located in the same domain. In contrast, transfer learning is not limited to samples in the same domain but can be applied to slightly related categories. Even completely unrelated samples are possible, but additional learning methods need to be designed to deal with the data mismatch problem.

For the above reasons, the challenge of applying general deep neural networks to the field of automatic optical inspection is the lack of enough datasets for network training. Each company has a different deployment to use this inspection technology inside the production line; this also differs from the datasets for different components. To detect each product well, it needs to take a large number of datasets and even label each data point, which is a very time-consuming and costly process. In contrast, the learning method used for transfer learning does not require a large dataset of objects to be examined, but only the design of a learning model that fits the current application situation. Finally, the network is trained with the rest of the different but related data, thus eliminating the need to produce large datasets.

In the method based on transfer learning, both [10,11] use a deep neural network based on VGG-16 to train the model for detecting optical images. VGG-16 is a network model that uses a large amount of data to train and has many layers inside. This method trains the parameters and weights inside the source domain network with the 1000 classes provided by ImageNet, and transfers all of the weights from the trained model to the convolutional layers of the target domain network through a designed transfer method. Then, the target domain convolutional neural network is fine-tuned by the dataset to be used for detection, e.g., [10] trains the network with six different types of texture patterns, and the trained network model detects which of the input texture patterns has defects. In [11], the network is trained by inputting different types of battery weld images and detecting which of the input images has a defective battery weld.

In addition, there are still some automatic optical inspection methods that use a non-transfer learning architecture [12,13,14]. In [15], a deep convolutional neural network is trained to detect scratches on the optical fiber connector end-face. The model consists of three main blocks, namely the feature extraction network for feature extraction, the BFE module with the CCMP block, and the SAU module. First, the image and ground truth are input into the network model, and the features are extracted by convolution. The boundary characteristics of the defect are then refined by a specially designed BFE module. Finally, the SAU module is used to locate and cut out the defect and output the defect location in the image.

### 2.2. Methods of Rule-Based Feature Extraction

In the existing methods, rule-based methods on feature extraction are widely used such as [16,17,18]. Although they are not more accurate than deep neural networks trained with large datasets, this technique is still needed due to its low complexity, low hardware requirements, and fast execution speed. Compared to deep neural networks, which train and update weights to obtain various features from a large number of datasets, traditional rule-based feature extraction methods take different approaches to capture features depending on the object.

For some mainstream detection and tracking fields, the optical flow method, frame difference method, and background subtraction method are adopted. The optical flow method determines the object’s position by calculating the object’s motion trajectory, while the frame difference method calculates the change of pixels in the front and back frames to find the object in motion. For the background subtraction method, Barnich proposed an innovation classification model technology, named ViBe [19]. This method uses several frames of images as background to adapt to complex dynamic environments. Although some AOI methods use this technique to detect objects in motion or changes in the surface of an object [20], it is still not useful for this paper being used to detect static objects.

In [21], the wire harnesses in the image were detected by using traditional feature extraction. First, they carried out a series of pre-processing on the incoming image such as noise reduction, contrast enhancement, etc. After that, the wire harness was divided into four interesting blocks by individual design, which were the whole line body, the color part of the line head, the QR code, and the black square component in the middle. Since the objects to be detected in this paper were all the same type of wire harness, including the color and placement of the wire, it was possible to detect the area of interest by specially designed color masking. Finally, the wire length, color composition, etc. were calculated from the blocks obtained previously, and the results were displayed on the screen.

In [22], an AOI system based on simple feature processing was used to inspect railway sleepers and surface defects. As the number of defective railway sleepers was very small compared to the number of non-defective railway sleepers, it was more difficult to adopt a machine learning approach. Firstly, this paper calculated the derivatives quickly through Haar transform and integral images and found the position of the rail in the image through a single Haar structure. Similarly, the fasteners were located by rotating the Haar structure. To accurately identify sleepers, the low entropy area of the image was searched first, and then the correct sleeper location was filtered out by Sobel vertical edge features. Once the correct position information was obtained, a specially designed method could be used to detect if a railway sleeper had a defect.

### 2.3. Common Wire Types

In this paper, the object to be inspected is a wire. However, there is a huge variety of cables, such as the ones used as USB, network cables, telephone cables, HDMI, etc. Each different cable has a different internal composition. Among all types of cables, most of them are multi-core wires made from multiple single-core wires, so most wire manufacturers need to check the correct color alignment of multiple single-core wires inside the cable during the fixing stage to ensure the normal function of the cable.

The most common type of wire inspection is a single-core wire with only one color. Another common cable is used in the RJ45 Ethernet connector, and it is also a single-core wire but consists of one or two colors in its wrap. In addition to the above two types of single-core wires, USB Type-C has become increasingly popular in recent years, and this cable is composed of multiple single-core and multi-core wires. For this multicore wire inspection method, the color of the aluminum foil wrapped around the wire and the color of the wrapped wire should be considered at the same time. For the convenience of reading, the single-core wire with only one color, the single-core wire with two colors, and two wires covered with aluminum foil are abbreviated as single-color wire, two-color wire, and multicore wire, respectively.

## 3. Proposed Method

**Figure 2:**
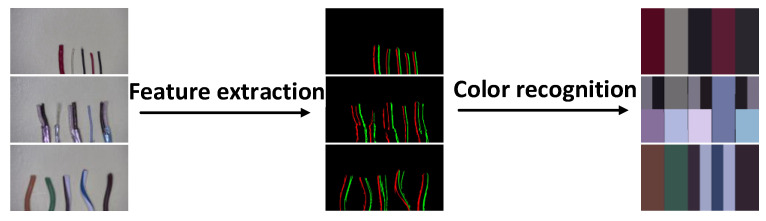
Basic architecture based on feature extraction.

Since the main types of wire used today are single-color wire, two-color wire, and multicore wire, these three types are the main objectives detected in this paper. The basic architecture of the system is shown in Figure 2. The whole system architecture with the related function is divided into two major parts: feature extraction and color recognition. First, a series of aligned wires are arranged, and the edge features of each wire in the image are captured by the feature extraction method designed in this paper. Once the edge features in the image are obtained, a specially designed discriminator is used to identify the color placement of the wires in the image from left to right. The following two parts of feature extraction and color recognition are explained separately.

### 3.1. Feature Extraction

**Figure 3:**
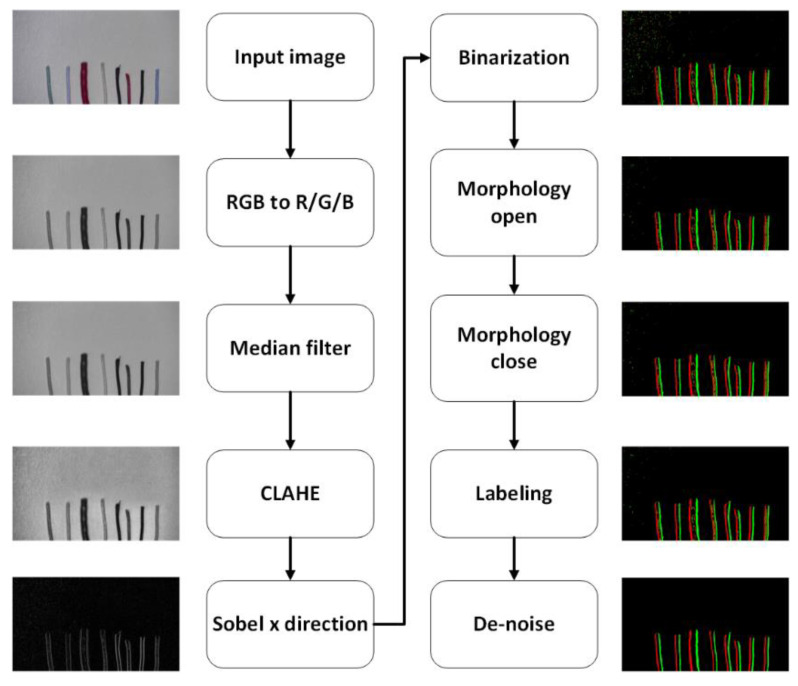
Flowchart of feature extraction.

Figure 3 shows the feature extraction method used in this paper. Due to the wide range of colors of the wire, using only black and white images or other single-color information for processing makes it difficult to distinguish some colors of the wire. Similarly, if the background colors of different colors are calculated by a single-color domain, it will make the accuracy of some background colors worse. For the above reasons, to obtain more image information of different colors to further distinguish the location of different wires, this paper converts the incoming images into R, G, and B domains for respective calculations.

#### 3.1.1. Pre-Processing

In image processing, to further improve the overall image quality, noise reduction is often performed to avoid the effects of noise. To improve the quality of products, companies often use high magnification and high-resolution digital microscopes to check the status of products. When taking pictures of wires in this way, the surface wrinkles of the wires and even the noise in the background become particularly obvious. To solve this situation, this paper adopts the median filter to reduce the noise in the overall image. This method replaces the original pixels by expanding each pixel in the image outward by a certain range and arranging the values in the range from smallest to largest to find the middle value. By using a median filter, it has the advantage of fast and effective noise reduction, as well as the ability to maintain the edge features without too much blurring. This issue is particularly useful for the expected edge computation of this system.

In addition, since the types and colors of wire used in practice are very diverse, it may not be easy to distinguish between the background and the color of the wire because they are too similar. To deal with this situation, a more common method is to directly use histogram equalization (HE) to enhance the overall contrast of the screen. This method calculates the number of times in each pixel brightness value appearing in the whole image and equalizes all the pixels appearing in the image. The final result is a more consistent number of times for each pixel brightness value throughout the image. The advantage of HE is that it is easy and effective to enhance the overall contrast of the screen so that some pixels with too similar colors are separated from each other. However, the disadvantage of this method is that it calculates the distribution of all the pixel intensity values on the whole screen. It can result in images with some feature information concentrated in a small area or images that are regionally brighter or even darker being forcibly scattered and thus creating broken blocks.

In the factory, to identify the color sequence of the wires, employees usually place multiple wires flat on a clean worktable. Therefore, the background occupies a large part of the image, it is not suitable to use histogram equalization to process. To deal with the drawbacks of this method, this paper uses contrast-limited adaptive histogram equalization (CLAHE) [23,24] to enhance the local contrast. Compared to the normal HE method, which calculates the distribution of pixel intensity values over the whole screen, this method only calculates the distribution of pixel intensity values between a pixel point and its surrounding area. The value of the pixel is then obtained by using the transform function derived from each histogram to enhance the contrast and feature information of the local area.

**Figure 4:**
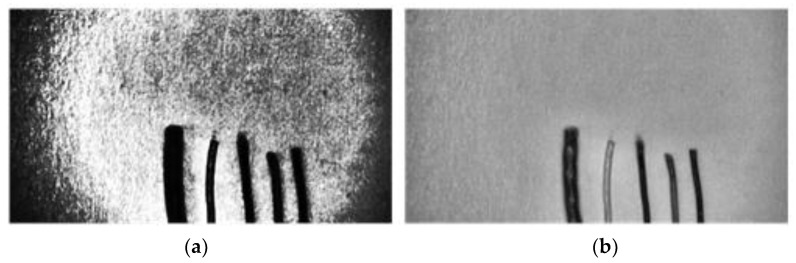
Contrast adjustment for different methods. (**a**) HE. (**b**) CLAHE.

However, only enhancing the local contrast still cannot eliminate the effect of too bright and too dark blocks on the image. Therefore, the contrast between too dark and too light areas is limited by cropping the overly concentrated areas in the histogram and redistributing them equally in the histogram before calculating the cumulative distribution function, as shown in Figure 4. The original HE algorithm enforces enhanced contrast in the background part that occupies most of the area, resulting in serious background brokenness. By limiting the contrast adaptive histogram equalization method, the overall background part is relatively intact, while the contrast of the wire edges is significantly enhanced.

#### 3.1.2. Sobel Operation

In this paper, it is important to select features that clearly distinguish between wire and non-wire to recognize the wire color later. There are many types of features in image processing, such as low-level features, curvature, image motion, shape correlation, etc. Optical flow is a common feature used to detect moving objects on the screen, while Hough transform is more commonly used to identify straight lines, circles, or even ellipses in the image. In this paper, the system is designed to identify the color sequence of the lines. Although the Hough transform can detect the location of straight lines in the entire image, not all the wires captured in the actual situation are straight and without curves. Therefore, after testing and experimentation, edge detection in the low-level feature category is finally adopted. This method is effective in capturing information about the edge locations with high contrast throughout the image, not limited to any shapes.

We use the Sobel operator as the basic method for edge detection to maintain a high processing speed [25,26]. The equation is shown below:

$$G_{x}=\left[\begin{array}{ccc} +1 & 0 & -1\\ +2 & 0 & -2\\ +1 & 0 & -1 \end{array}\right]*A$$

$$G_{y}=\left[\begin{array}{ccc} +1 & +2 & +1\\ 0 & 0 & 0\\ -1 & -2 & -1 \end{array}\right]*A$$

$$G=\left|G_{x}\right|+\left|G_{y}\right|$$

where A represents the original input image, G is the gradient magnitude calculated from the horizontal gradient $G_{x}$ and the vertical gradient $G_{y}$. In this paper, since the object to be identified is the vertically placed wire, the horizontal gradient is more meaningful than the vertical one. To enhance the feature information, the proportions of $G_{x}$ and $G_{y}$ are reassigned through the weights $w_{x}$ and $w_{y}$, as follows:

$$G=w_{x}\left|G_{x}\right|+w_{y}\left|G_{y}\right|$$

**Figure 5:**
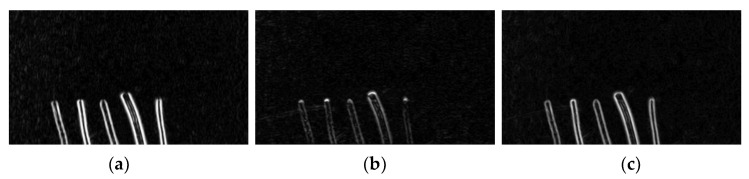
Impact of different weights on features. (**a**) *w_x_* = 0.9, *w_y_* = 0.1. (**b**) *w_x_* = 0.1, *w_y_* = 0.1. (**c**) *w_x_* = 0.5, *w_y_* = 0.5.

Increasing the gradient weight in the horizontal direction can increase the clarity of the edge features of the wire on the one hand. It can also reduce the effect of some wrinkles or irregular shape noise on the wire on the other hand. As shown in Figure 5, the larger the percentage of $w_{x}$, the more obvious the overall edge features of the wire. When the percentage $w_{y}$ becomes larger, the edge features of the wire become quite invisible, and only the part of the wire head has more obvious features. For $w_{x}$ and $w_{y}$ with similar proportions, the result is in between, and some noise becomes clearer compared to the other two.

When the input image is calculated above, the gradient magnitude of each pixel is obtained. After that, the pixels with gradient values greater than a certain threshold are determined to be true edges by binarization, and the other pixels are considered noise and removed. In addition, for the further distinction of the different wires, all the obtained edge features are determined in two types according to the following method, which is designed to distinguish the gradient in different directions:

$$Feature\left(x,y\right)=\{\begin{array}{c} Edge\;1,\;if(G_{x}<0)\\ Edge\;2,\;otherwise \end{array}$$

#### 3.1.3. Mathematical Morphology

There are inevitably some fracture features in the obtained edge features, and these are probably caused by insignificant edge features leading to breaks in the middle part of the wire. In addition, some shadows, noise, and even wrinkles in the wire result in incorrect edge features after the calculation. For the above reasons, this paper adopts Opening and Closing to deal with this problem [27,28]. The Opening process is calculated by first doing the erosion and then the dilation, while Closing process is the opposite.

**Figure 6:**
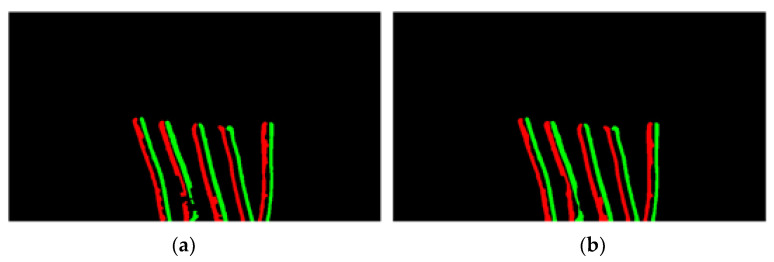
Impact of the different kernel on features. (**a**) Square kernel. (**b**) Rectangle kernel.

Firstly, the system removes some undesired edge features and noise by Opening computation to make the overall edge structure smoother and then reconnects some broken features by Closing computation to make all edge features more coherent. The advantage of using these two methods is that they eliminate noise and connect broken features without destroying the original edge features too much. To further restore the broken edge feature, a vertical rectangle is used as the kernel for the Closing operation. This design can effectively connect the edge features of the wire in the same vertical direction, as shown in Figure 6. The rectangular kernel has a more consistent edge feature, while the square kernel still has a broken edge feature of the wire.

#### 3.1.4. Object Labeling

This inspection is done under a high magnification, high-resolution digital microscope, so additional light sources are often set up to illuminate the wire for easy observation. Since the surface material of the wire is not particularly smooth and easy to reflect, the wrinkles of the wire surface and the reflections caused by the light source are also magnified several times in the image. Both the shaded areas of wrinkles and the bright areas of reflections have very high contrast and occupy a large area relative to the surrounding pixels in the image, these unwanted additional features are retained after the previous series of processing.

**Figure 7:**
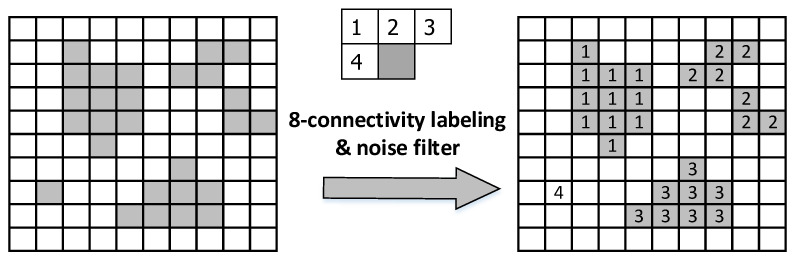
The 8-connectivity based labeling.

To further eliminate the additional effects mentioned above, this paper specifically adopts the 8-connectivity-based labeling method [29,30]. By distinguishing all adjacent edge features in the same category, all pixels are labeled separately and the pixels in the same label are the same edge feature. Figure 7 shows the specific processing results. By labeling the left figure with 8-connectivity-based labeling, four labels are generated and each label represents an unrelated object. In addition, this method checks the eight surrounding directions, so the results are more continuous.

#### 3.1.5. De-Noise

**Figure 8:**
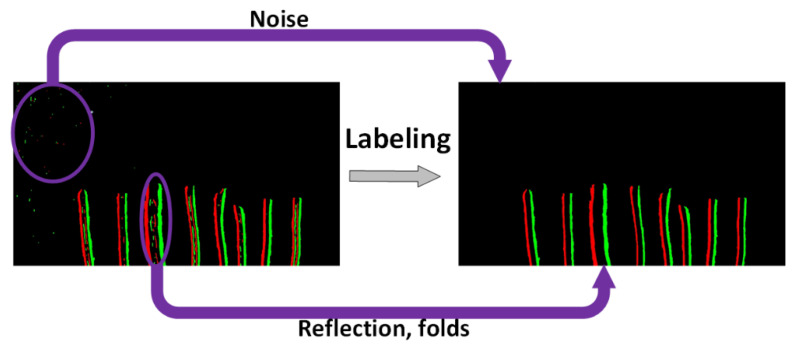
Results of eliminating redundant features.

After obtaining the labels of all the features, the size of the area occupied by each object in the image can also be calculated accordingly. For this paper, the object that occupies the largest area of the image is used as a standard to further reduce redundant features. In this inspection environment, the wire is the only object that appears in the image, so the largest feature that appears in the image should also be the edge feature of the longest wire in the image. In addition, the folds on the surface of the wire or the reflections from light are relatively thin features on the wire, and the length is no longer than the longest wire. The edge feature of the longest wire in the input image is determined by the label type that occupies the largest area, and a certain range is set for noise removal based on this. Label types within this range are judged to be wire edge features, while label types outside of this range are consistently judged to be unwanted redundant features such as noise, folds, reflections, etc. As illustrated in Figure 8 after the labeling, edge features that occupy the largest area are used to eliminate such as noise, thin reflections of light on the wire, and even broken surface folds.

The final result is a clean background with smooth and continuous edge features for each wire.

### 3.2. Color Recognition

**Figure 9:**
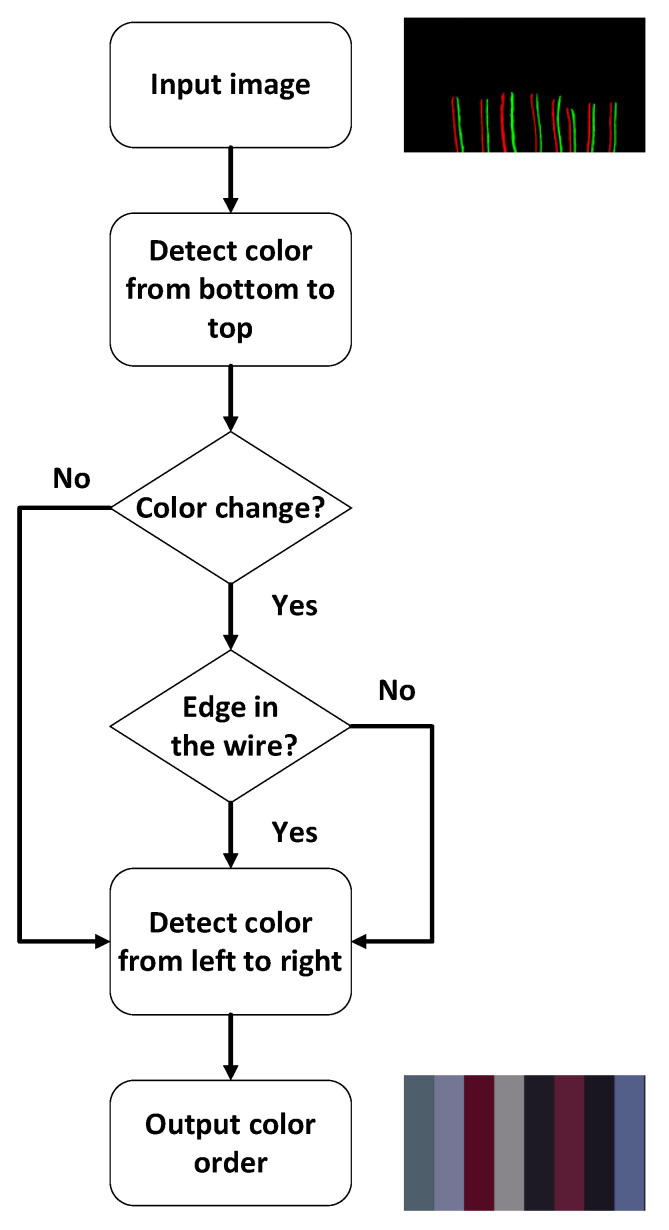
Flowchart of color recognition.

With the edge feature maps extracted in the previous section, this section discusses in particular the color recognition method adopted. Figure 9 shows the flow chart for detecting the color sequence of the wires used in this paper. Firstly, after the edge features generated in the previous stage are input, the system detects this input from the bottom of the image upwards. Since the background is a fixed color, the edge features of the left and right sides of the wire should be in different gradient directions. During the scanning process, it is possible to determine the location and color composition of each wire from left to right and to detect whether each wire changes color midway. If there is no color change in the middle of the process, it is assumed that this wire is the most basic single-color wire and the color of each wire is output from left to right. If a color change is detected in a wire during the scanning process, the wire is further detected by an additional discriminating mechanism.

The first step is to determine if there is an additional edge feature in the middle of the wire. Assuming that no additional edge features are observed, it is judged that the wire is a different color wire at the top and bottom and output the color composition of each wire from left to right. When an additional edge feature appears in the middle of the wire, the output differs depending on whether the additional edge feature appears suddenly from the middle of one of the left or right sides. If it appears suddenly from the middle, the wire is judged to be one color at the lower end, and two wires are wrapped at the upper end. If the wire is connected from one of the left or right sides, it is judged to be a combination of two colors similar to the RJ45 Ethernet connector. Finally, for the observation purpose, the result is output as a color block diagram in Figure 9.

## 4. Experimental Results

### 4.1. System Specification and Image Resolution

**Figure 10:**
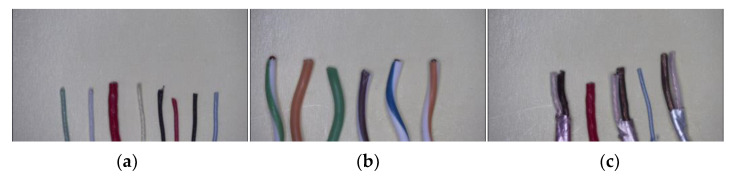
Different types of wire. (**a**) Single-color wire. (**b**) Two-color wire. (**c**) Multicore wire.

The recognition environment is simulated and evaluated by using C++ on a computer with a 4 GHz Core i7 CPU, and the selected inspection tool is the AD409 digital microscope produced by Andonstar. The resolution of the output image is 4032 × 3024, and the magnification can reach 240 times under the 27-inch screen when the distance is 5 cm. The test wire is the ordinary single-color wire, two-color wire used in RJ45 Ethernet connector, and multicore wire used in USB Type-C, respectively, as shown in Figure 10. Their diameters are 0.511 mm, 0.511 mm, and 0.812 mm, respectively. These three types of wire are chosen because there are many different types of wire in the factory. In the following evaluation, we will discuss some factors which influence the detection result of the system.

Firstly, to verify the effectiveness and timeliness of the system, Table 1 shows the effect of different image resolutions on the accuracy and execution time of the system. In this experiment, the image resolution is standardized to 4:3 and scaled in proportion, and the maximum resolution is limited by the use of a digital microscope of 4032 × 3021. The average accuracy in the table is determined by directly comparing the color sequence of the input image and the output result. If the comparison is completely correct, the output is judged to be correct; otherwise, the output is wrong. In addition, for consistency, a single-color wire is used for comparison. The background is unified in white, and the wire is exposed from the bottom by 5 mm. The entire dataset contains 200 images of the scenarios described above, and each image contains three to six wires.

When the image resolution is higher, the average accuracy increases as the overall feature information increases. However, once resolving 2048 × 1536 or even 4032 × 3024, the average accuracy decreases rather than increases. This is because when images are taken at high resolution, things such as noise or folds and reflections on the surface of the wire are also overly magnified. Due to these unexpected features, the average execution time also increases significantly when the resolution reaches 2048 × 1536 and it becomes more difficult to keep the system running in real-time. After this experiment, it is believed that the resolution of 1600 × 1200 or 1280 × 960 has better accuracy and the execution speed can be run immediately, so the following experiments are run with the resolution of 1280 × 960.

### 4.2. System Profiling and Result

**Figure 11:**
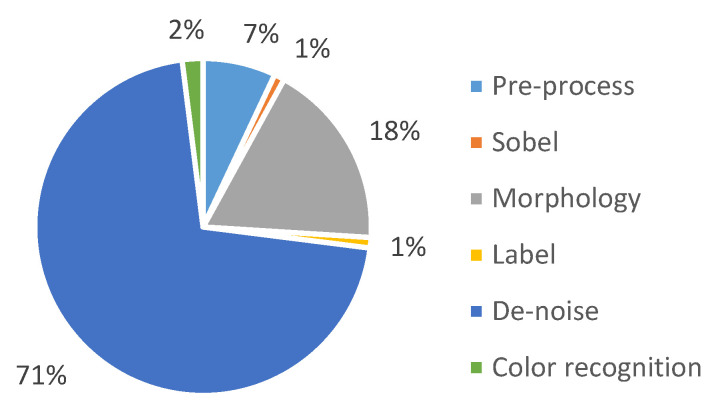
Execution time required for different stages.

Figure 11 shows the time required for each stage of the system. It can be found that the longest time is required for de-noise. The percentage of time spent in this stage grows as the resolution increases, which means that the high resolution also generates a lot of noise.

**Figure 12:**
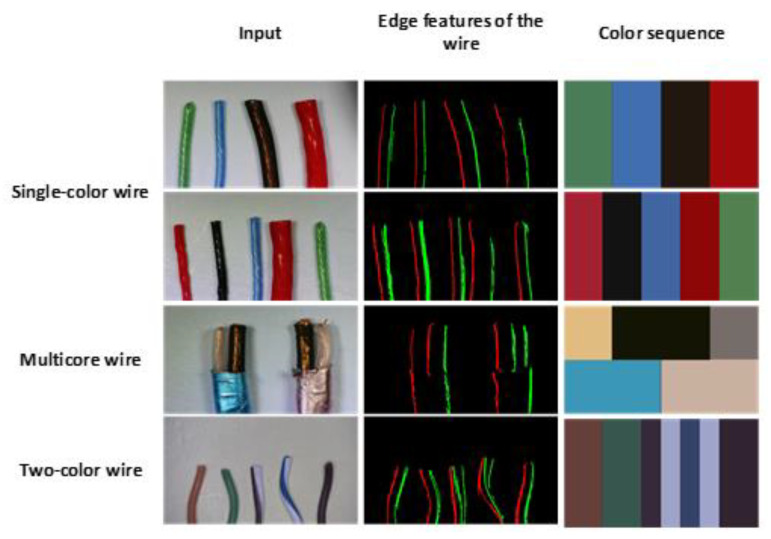
Output results of different wire types.

Figure 12 shows the results of feature extraction and color sequence of the system for different wires. For the convenience of the display, different colored edge features represent different gradient directions, and the output has colored blocks from left to right to represent the color of the wires in the input image from left to right, respectively. The multicore wire is divided into upper and lower parts, while the two-color wire splits the original block into two pieces.

To simulate the operation of the production line in the real factory and further verify the integrality of the system, the test results are done by combining different wire materials and different color backgrounds with each other as shown in Table 2. In this experiment, 50 images are taken from each group of test situations and run at 1280 × 960 resolution. For different wire types, it is clear that the accuracy is highest in the case of single-color wire followed by multicore wire, while the accuracy of two-color wire is much lower than in the other two cases. This result is also easily understood even if the detection task is made by humans. The technical reason is that the wire is twisted to make two colors appeared in one plane, and the maximum area that the digital microscope can contain may not be able to appear in two colors at the same time. When taking pictures of the dataset, due to the angle of placement, it is easy to have only one color appearing on the surface of the wire, resulting in a loss of accuracy.

For different background colors, white backgrounds generally show relatively good results. Only in the case of two-color wire, the accuracy of the black background is higher than that of the white background. This is because the two-color wire is a white part with another color, which causes the white part to be ignored due to low contrast after capturing the features on the white background. In addition, the white and black backgrounds are the very large and very small values of the grayscale values, which are more convenient for the system to recognize edge features, and the yellow background is in between. This may lead to misjudgment of the edge feature when identifying the multicore wire that has different colors on top and bottom.

### 4.3. Evaluation for Different Wire Lengths

To further test various situations in the factory, the length of the wire exposed in the image is additionally adjusted and the results of the experiment are recorded in Table 3. For multicore wire, since both upper and lower parts of this wire need to be inspected, the upper and lower sections are cut at the same time for inspection. Due to the above reasons, it is difficult to divide this wire into five situations like single-color wire, so only 5 mm and 2.5 mm are used for the test. In the case of two-color wire, it is more difficult to display both colors under a high magnification digital microscope, so there is no way to shorten the wire. Based on the mention above, only single-color wire and multicore wire are tested in this experiment.

In this experiment, 100 images are prepared for different exposure lengths and run with a resolution of 1280 × 960 with a white background. The length of the wire should affect the execution time since the length of the wire is proportional to the detection region. When the exposed length of the wire is more than 3 mm, the average accuracy remains at a good level. When the length exceeds 5 mm, the effect of improvement becomes limited. However, once the exposed length is only 2 mm, 1 mm, or even 2.5 mm, the accuracy rate drops significantly. This is because the shortening of the wires in the image will cause the overall edge features smaller. This system takes the longest edge feature as the basis when dealing with wrinkles and reflections on the surface of the wire, so this kind of noise is not easily eliminated with shorter edge features.

## 5. Conclusions

In this paper, an AOI system for detecting color sequences of wires based on rule-based feature extraction technology is proposed. By generating the clean edge features of each wire at the front end, the color composition of the wire is later identified by a specially designed discrimination mechanism. In the whole process, the system adopts a low-computation and low-complexity processing method as much as possible, so that the optical inspection system can run immediately in most environments. In addition, the special discrimination mechanism in our design can allow the system to detect several common types of wires instead of regular single-color wires. By capturing features in this way, the accuracy can be maintained even at lower resolutions or with shorter wire exposure. Due to the convenience and accuracy of this system, it can effectively replace the excess labor cost based on a single camera with a computer vision technique to achieve the goal of automatic optical inspection.

## Figures and Tables

**Table 1:** Results of different image resolutions.

Image Resolution	Average Accuracy (%)	Average Time (s/frame)
4032 × 3024	89	18.934
2048 × 1536	91.5	4.126
1600 × 1200	98.5	1.686
1280 × 960	98	0.972
1024 × 768	91.5	0.548
800 × 600	93	0.248
640 × 480	92	0.198
480 × 360	89.5	0.094
320 × 240	75.5	0.051

**Table 2:** Results of different background colors and different types of wires.

Wire Type	Background Color	Average Accuracy (%)
Single-color wire	White	98
	Black	96
	Yellow	94
Multicore wire	White	82
	Black	80
	Yellow	74
Two-color wire	White	62
	Black	72
	Yellow	62

**Table 3:** Effect of different lengths of wire exposure on different wires.

Wire Type	Wire Length (mm)	Average Accuracy (%)
Single-color wire	5	99
	4	98
	3	93
	2	81
	1	72
Multicore wire	5	83
	2.5	64

## Data Availability

Not applicable.
